# Four Recombinase-Aided Amplification Assays for the Rapid Detection of Fowl Adenovirus Serotype 4

**DOI:** 10.3390/ijms27219756

**Published:** 2026-10-30

**Authors:** Qixian Feng, Shaohua Lu, Yuting Cao, Ruisen Wu, Yuan Wang, Lihui Xu, Quanxi Wang

**Affiliations:** 1 College of Animal Science, Fujian Agriculture and Forestry University, Fuzhou 350002, China; feng.qixian@fafu.edu.cn (Q.F.); ls991020@126.com (S.L.); 12406008014@fafu.edu.cn (Y.C.); 52306023060@fafu.edu.cn (R.W.); 52306023003@fafu.edu.cn (Y.W.); 000l813037@fafu.edu (L.X.); 2 Fujian Key Laboratory of Traditional Chinese Veterinary Medicine and Animal Health, Fujian Agriculture and Forestry University, Fuzhou 350002, China; 3 University Key Laboratory for Integrated Chinese Traditional and Western Veterinary Medicine and Animal Healthcare in Fujian Province, Fujian Agriculture and Forestry University, Fuzhou 350002, China

**Keywords:** fowl adenovirus serotype 4, recombinase aided amplification, lateral flow dipstick, clusters of regularly spaced short palindromic repeats, diagnostic method

## Abstract

Fowl adenovirus serotype 4 (FAdV-4) infection leads to serious damage to the poultry industry worldwide. Accurate and prompt diagnosis is essential for effective management of FAdV-4 outbreaks. In this study, we first designed a pair of specific primers based on the *ORF19A* gene of FAdV-4 strain NP for recombinase-aided amplification (RAA) and optimized the reaction conditions. The results revealed that the optimal conditions for a sensitive RAA reaction were at 39 °C for 40 min. The minimum detection amount of FAdV-4 genomes was 10^−2^ ng/μL using RAA. In order to facilitate the result presentation of RAA, which was visualized using a lateral flow dipstick (LFD), and the optimal reaction time of RAA-LFD was shortened to 30 min. However, the minimum detectable amount of FAdV-4 genomes was 50 ng/μL, which was lower than the sensitivity of RAA. Furthermore, to enhance the sensitivity of RAA-LFD, we designed LbaCas12a-FAdV-4-crRNA and incorporated it into RAA to develop the RAA-Cas12a-F and RAA-Cas12a-LFD methods, respectively. Based on the RAA reaction of 40 min, RAA-Cas12a-F and RAA-Cas12a-LFD only need an additional 15 min. The minimum detectable amounts of RAA-Cas12a-F and RAA-Cas12a-LFD were 10^−6^ ng/μL and 10^−5^ ng/μL, respectively. These results indicate a significant improvement in the sensitivity of two detection methods. A total of 192 fecal samples from chicks were collected from Fujian province. These samples were then detected using these four RAA rapid detection methods. The results showed that 57 positive samples were screened by both RAA-Cas12a-F and RAA-Cas12a-LFD, which was 4 more positive samples than the RAA method and 14 more positive samples than the RAA-LFD method. Overall, we developed four accurate and rapid RAA assays of FAdV-4, which is feasible and of certain value in the application of FAdV-4 in clinical practice.

## 1. Introduction

FAdV-4 causes huge economic losses to the global poultry industry [1]. Based on restriction endonuclease digestion and serum cross-neutralization tests, FAdV was classified into five groups (FAdV-A~E) and 12 serotypes of FAdV group A (FAdV-1~8a,8b~11) [2]. The capsid of the virus is composed of major structural proteins, including fiber, penton, and hexon [3,4]. Fiber 1 increases the binding rate of the receptor on the cell membrane to wild strains [5] and requires the participation of importin α1 for its entry into the nucleus [6]. Fiber 2 affects the proliferation process of the virus and enhances the production of neutralizing antibodies [7,8,9]. Though many studies have explored the pathogenic mechanism of FAdV-4 infection, it is important to establish a rapid and accurate detection technology for quickly controlling the virus epidemic [10].

RAA represents a novel isothermal nucleic acid amplification technology demonstrating broad application potential in pathogen diagnostics. This innovative approach employs a specialized enzyme cocktail comprising three core components: single-stranded DNA-binding protein (SSB) for strand stabilization, UvsX recombinase mediating strand invasion, and DNA polymerase enabling subsequent strand extension [11]. At a constant temperature, the recombinase tightly binds to the primers, forming an aggregate of enzymes and primers. With the assistance of SSB, the template DNA is unwound, and new DNA complementary strands are formed under the action of DNA polymerase. Typically, the reaction is performed at 37 °C to 42 °C for 0.5 h to 1 h [12]. Compared to PCR, RAA offers the advantage of evaluating results without relying on costly instruments and equipment. It is suitable for non-laboratory settings requiring the analysis of numerous samples, and the convenience of room temperature amplification facilitates field diagnostics [13].

The clustered regularly interspaced short palindromic repeats/CRISPR-associated proteins system (CRISPR-Cas) is an adaptive antiviral immune system present in most archaea and bacteria. It functions through the principle of self-non-self-recognition [14]. It has been applied to nucleic acid detection by specifically binding to target sequences using single-stranded guide RNA (crRNA) [15]. The Cas12a protease relies on its incidental cleavage activity to detect the presence of specific DNA/RNA in vitro by cleaving non-target-labeled DNA. To determine the results of the assay, ssDNA incorporating fluorescent-biotin modifiers can be used. These results can be analyzed using lateral flow chromatography (LFD) test strips. Due to the specific recognition of target sequences by crRNA, CRISPR-Cas has higher specificity, equivalent to the secondary screening of nucleic acid amplification products. By combining the CRISPR-Cas12a system with RAA, the detection sensitivity and specificity of loop-mediated isothermal amplification (LAMP) would be significantly improved [16].

In this study, we compared the genomes of 12 different serotypes of FAdV and found that the ORF19A gene (Figure S1) of the FAdV-4 virus was more conserved. Based on the ORF19A gene, we designed specific primers and established a series of detection methods, including RAA, RAA-LFD, RAA-Cas12a-F, and RAA-Cas12a-LFD. These methods could provide a rapid, accurate, easy to operate, and sensitive detecting application for FAdV-4 in poultry industry.

## 2. Results

### 2.1. Establishment and Preliminary Application of RAA Assay for Detection of FAdV-4

**Figure 1:**
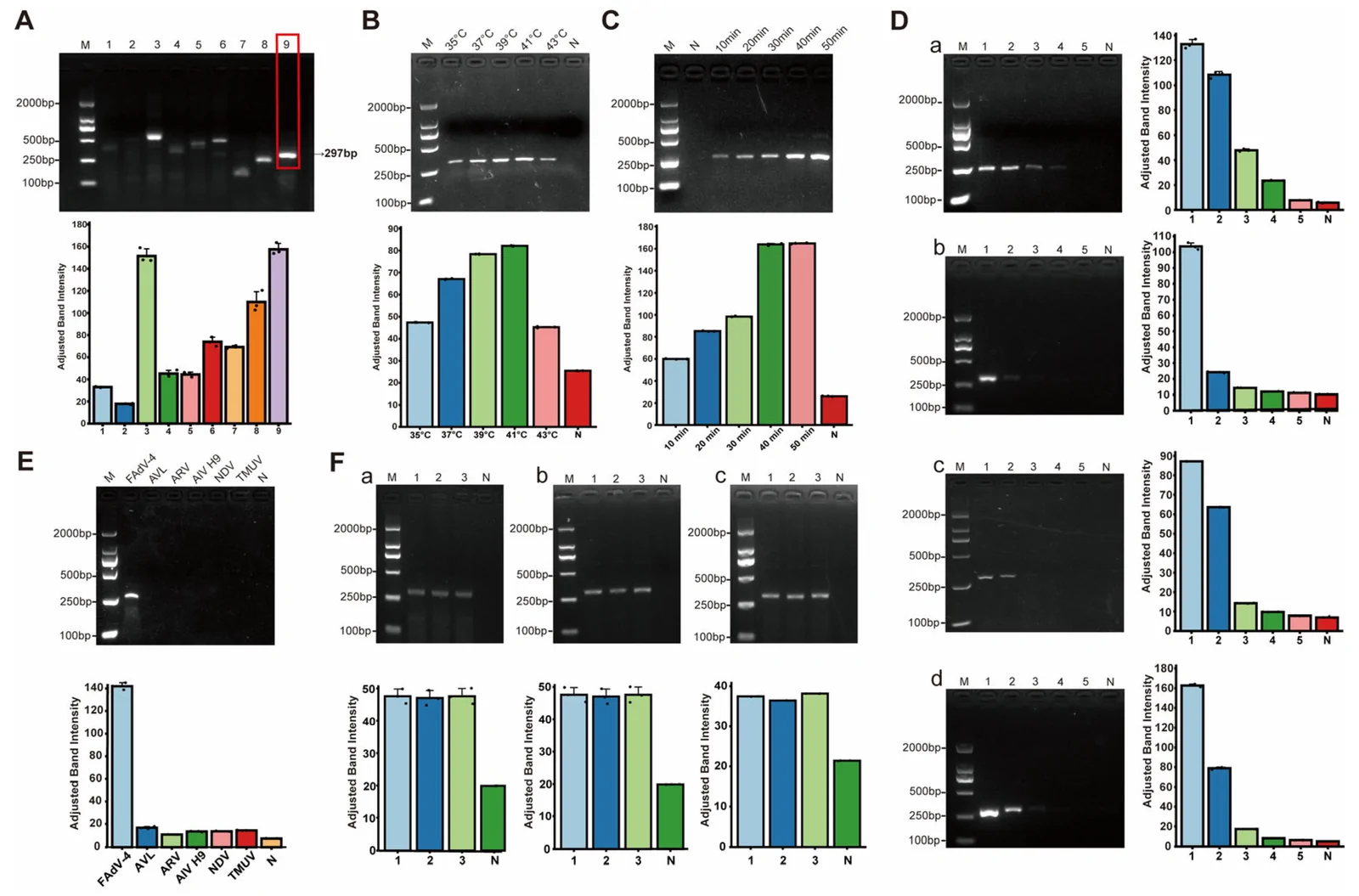
Development of RAA detection for FAdV-4. (**A**) Agarose gel electrophoresis results of RAA products with nine pairs of primers (Red box: selected primer sequences, lane 1–9: RAA amplified results with 1st–9th pairs of primers). (**B**,**C**) Products of RAA amplification at different reaction temperatures and times, respectively. (**D**) Comparison sensitivity of RAA and PCR detection ((**a**,**b**) sensitivity maps of RAA and PCR using FAdV-4 DNA as templates, respectively (lane 1–5: 10^1^ ng/μL–10^−4^ ng/μL); (**c**,**d**) RAA and PCR sensitivity maps using the pEASY-T1-ORF19A clone plasmid as the template, respectively (lane 1–5: 10^5^copies/mL-10^1^ copies/mL)). (**E**) Specificity test of RAA for FAdV-4 detection. (**F**) Reproducibility tests for RAA ((**a**) three reaction tubes in the same kit were repeated three times in the batch of positive samples; (**b**,**c**) three inter-batch replicates of positive samples within two kits produced at different times). The histograms of sample band intensity were quantified by ImageJ (version 2.3.0). Lane M: 2000 DNA marker; lane N: negative control.

As shown in Figure 1A, the amplified fragment in lane 9, which was located at approximately 297 bp, exhibited the clearest band and better specificity. Its brightness was the strongest, as quantified by ImageJ (version 2.3.0). Therefore, the primers for RAA-F9/R9 used in lane 9 were selected as the optimal primers for RAA detection of FAdV-4. The amplification product was sequenced, which indicates that the primers of RAA-F9/R9 are specific. With the increase in the reaction temperature, the brightness of the ORF19A product band amplified by RAA initially became brighter and then gradually became darker. There was no significant difference in the RAA amplification efficiency between 37 °C and 41 °C. Therefore, the reaction temperature for subsequent RAA determination was set at 39 °C (Figure 1B). As can be seen from Figure 1C, with the increase in reaction time, the brightness of the target band of RAA amplification gradually increases with the increase in reaction time. Under the reaction conditions of 40 and 50 min, the target band appeared clear and bright. However, when the reaction time was extended to 50 min, a non-specific band appeared faintly above the target band. Additionally, 40 min was more in line with the requirements of rapid detection time, so a shorter time was selected for the subsequent test.

The RAA reaction was performed using various concentrations of FAdV-4 viral DNA as templates. In a 50 μL RAA reaction system, 10^−2^ copies/μL of viral DNA (Figure 1D-a) was detected, which was higher than the lowest concentration detected by PCR (Figure 1D-b). Different concentrations of ORF19A cloning plasmids were used as templates for RAA and PCR. The minimum detection of recombinant plasmids was 10^3^ copies/μL (Figure 1D-c,d). Specificity test results showed that the RAA reactions for Newcastle disease virus (NDV), avian leukosis virus (ALV), avian reovirus (ARV), avian influenza virus H9 subtype (AIVH9), and Tembusu virus (TMUV) were all negative. Only the RAA reaction using FAdV-4 viral DNA as a template yielded a positive result (Figure 1E). The repeatability test showed that the brightness performance of RAA reaction bands was consistent within batches (Figure 1F-a) and between batches (Figure 1F-b,c).

**Figure 2:**
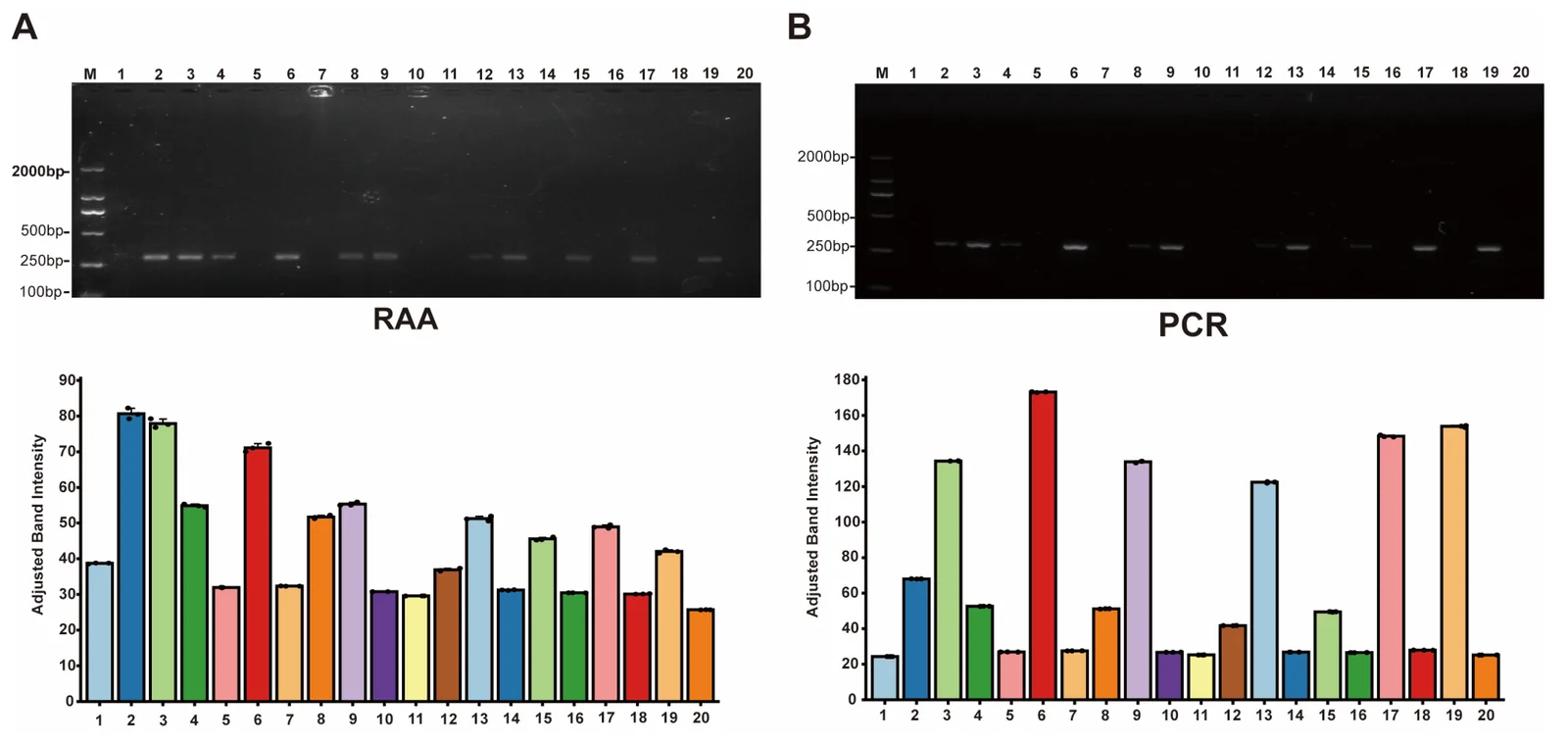
Detection of RAA (**A**) and PCR (**B**) in liver samples (lanes 1–20: different liver samples). The histograms of sample band intensity were quantified by ImageJ (version 2.3.0). Lane M: 2000 DNA marker; lane N: negative control.

Eleven clinical liver samples were found to be positive for FAdV-4 using both RAA and PCR methods, and the coincidence rate between the two methods was 100% (Figure 2).

### 2.2. Establishment and Preliminary Application of RAA-LFD Method for Detecting FAdV-4

**Figure 3:**
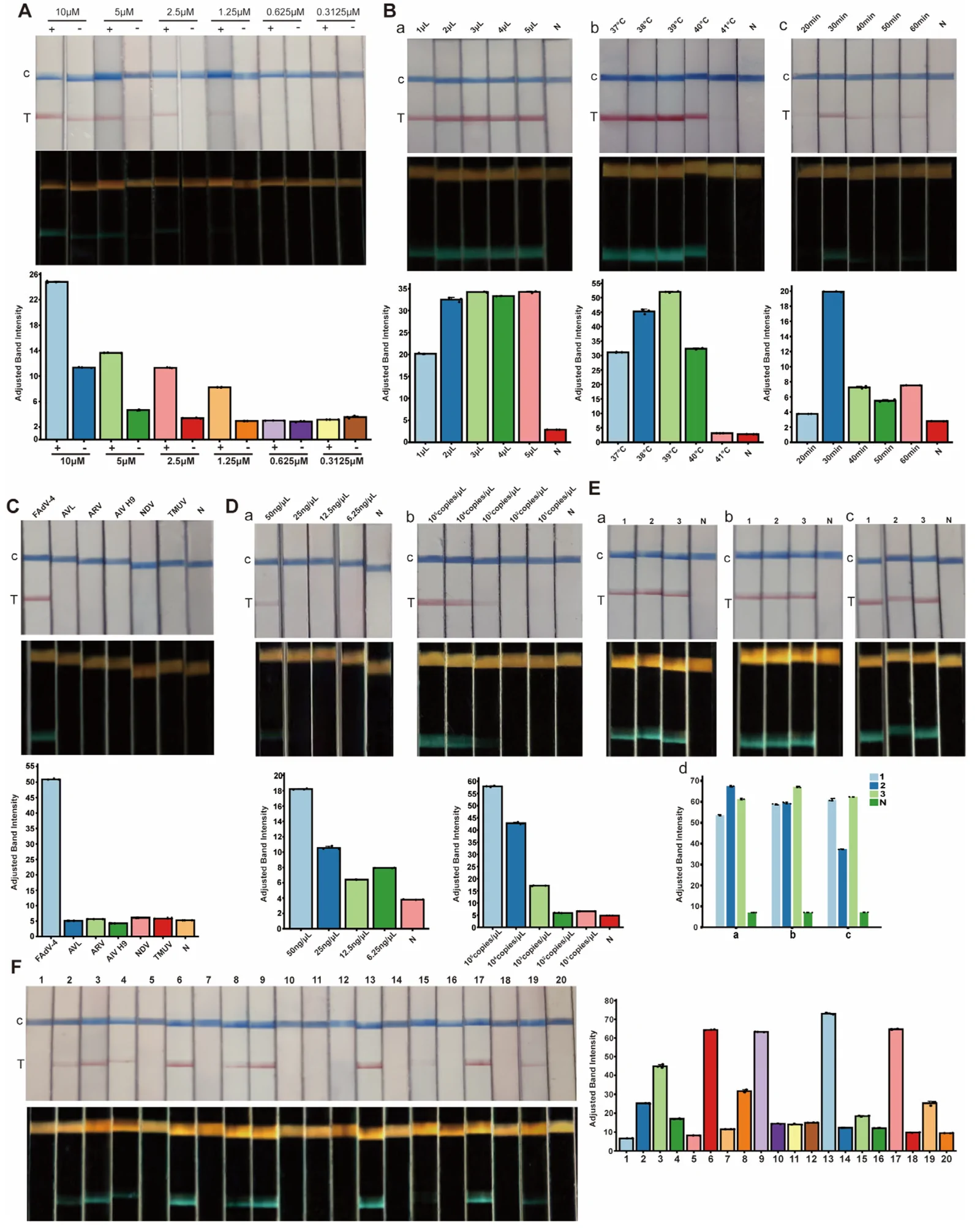
Development of RAA-LFD detection for FAdV-4. (**A**) Determination of RAA-LFD probe concentration. (**B**) Optimization of RAA-LFD reaction conditions ((**a**) template content optimization; (**b**) reaction temperature optimization; (**c**) reaction time optimization). (**C**) Specificity test of RAA-LFD for FAdV-4 detection. (**D**) Sensitivity test of RAA-LFD ((**a**) viral DNA was used as a template for detection; (**b**) pEASY-T1-ORF19A cloning plasmid was used as a template for detection). (**E**) Reproducibility tests for RAA-LFD ((**a**) three reaction tubes in the same kit were repeated three times in the batch of positive samples; (**b**,**c**) three inter-batch replicates of positive samples within two kits produced at different times); (**d**) The histograms of sample band intensity were quantified by ImageJ (version 2.3.0). (**F**) Detection of RAA-LFD in liver samples (lane 1–20: different liver samples). The image below the sample band, which has been converted to grayscale for quantification. N: negative control; C: control line; T: test line.

To facilitate the result judgment of RAA for FAdV-4, we developed the RAA-LFD method. The results showed that false positive results were observed on the T-line of the negative control strip at 10 μM and 5 μM concentrations of the primer and probe mixture, respectively (Figure 3A). When the concentration of the primer and probe mixture was 2.5 μM and 1.25 μM, the negative test strip with a concentration of 0.625 μM was correct, but the positive band also gradually became thinner. When the concentration of primer and probe mixture was 0.3125 μM, the positive test strip showed false negative results. Because positive results were the brightest and negative results were negative at 2.5 μM, a primer and probe concentration of 2.5 μM was selected as the most appropriate reaction concentration.

Figure 3B illustrates the reaction conditions of three different RAA-LFDs. The optimal template content of the FAdV-4 reaction system detection is 3 μL. The optimal reaction temperature is 39 °C, and the optimal reaction time is 30 min. The obvious color development of the T-line is used as the screening standard.

The specificity results showed that the RAA-LFD reaction results for ALV, ARV, AIVH9, TMUV, and NDV were all negative. However, the results for FAdV-4 viral DNA as a template showed obvious color T-lines, indicating a positive result (Figure 3C). This suggests that the method has good specificity. The lowest detection limit of the RAA-LFD assay was 50 ng/μL of FAdV-4 viral DNA. The lowest detectable level of the recombinant plasmid was 103 copies/μL in the RAA-LFD reaction system using the pEASY-T1-ORF19A cloning plasmid as a template (Figure 3D). The RAA-LFD method was used to test the intra-assay and inter-assay repeatability of positive samples. The results showed that the FAdV-4-positive samples detected by RAA-LFD were positive in both intra-assay (Figure 3E-a) and inter-assay (Figure 3E-b,c). It can be seen that the detection method exhibits high stability. The RAA-LFD assay showed that 10 samples tested positive for FAdV-4, resulting in a positive rate of 50%. Compared to RAA and PCR, the positive coincidence rate was 90.9% (Figure 3F). These results indicate that the RAA-LFD of FAdV-4 had lower sensitivity compared to RAA and PCR.

### 2.3. CRISPR-Cas12a for Detection of FAdV-4

**Figure 4:**
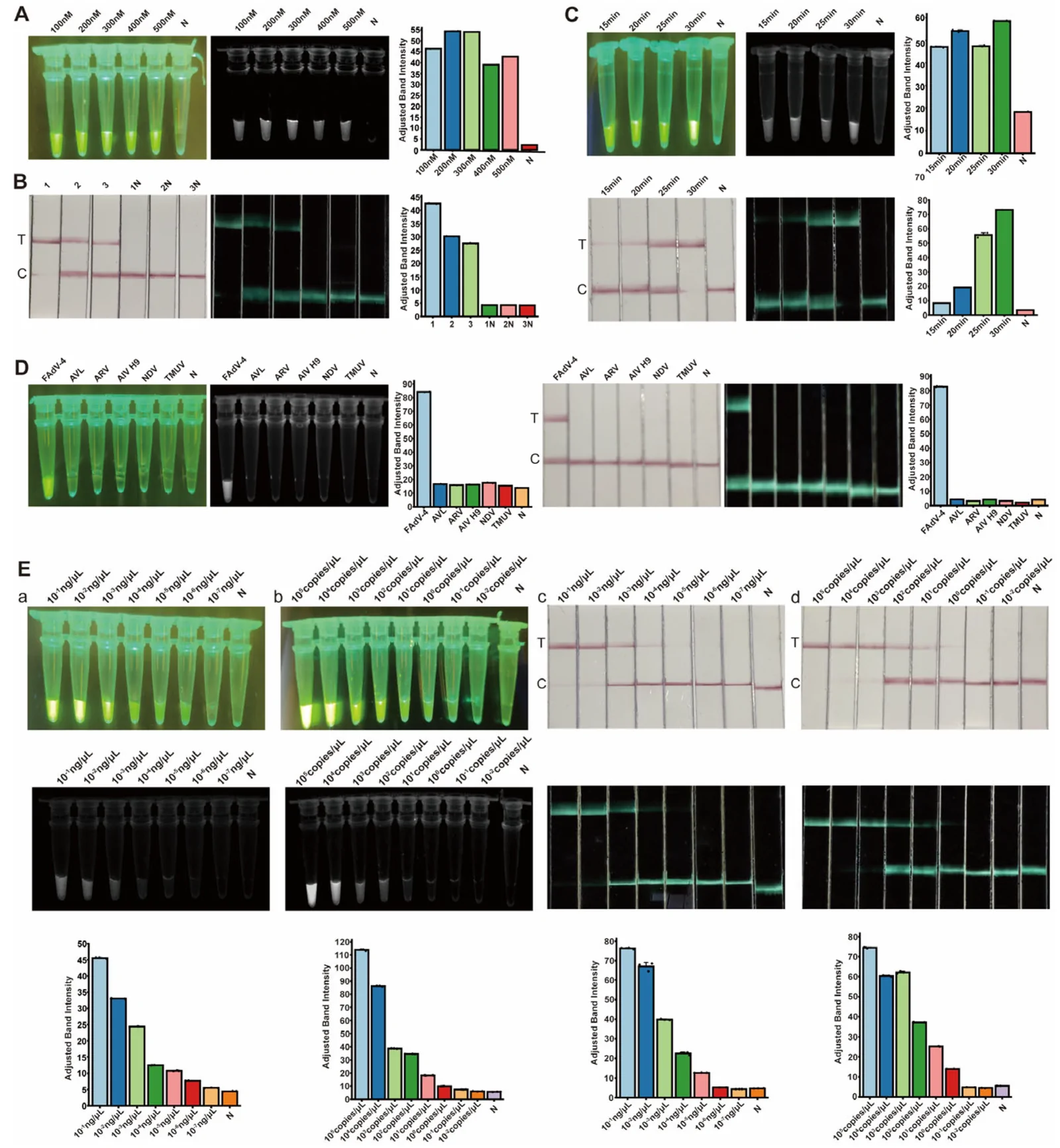
Establishment of RAA-Cas12a-F and RAA-Cas12a-LFD assays. (**A**) Results for different crRNA concentrations under blue light and UV, respectively. (**B**) Probe screening results (1: Fam−TTATT−Biotin; 2: Fam−TCCCT-Biotin; 3: Fam−TTTTTTTTATT-Biotin; 1N: Fam−TTATT−Biotin negative control; 2N: Fam−TCCCT-Biotin negative control; 3N: Fam−TTTTTTTTATT-Biotin negative control). (**C**) Optimization of reaction times for RAA-Cas12a-F and RAA-Cas12a-LFD assays. (**D**) Specific detection by RAA-Cas12a-F and RAA-Cas12a-LFD methods. (**E**) Sensitivity tests of RAA-Cas12a-F and RAA-Cas12a-LFD ((**a**,**c**) test results of different DNA nucleic acid concentrations; (**b**,**d**) test results with different plasmid concentrations). The results of the RAA-Cas12a-F assay were analyzed using images obtained under UV light and quantified with ImageJ. N: negative control; C: control line; T: test line.

In the present study, CRISPR/Cas12a was selected to improve the sensitivity and accuracy of RAA for FAdV-4. Figure 4A shows that the brightness of Cas12a was more pronounced when the crRNA concentration was 300 nM. Therefore, we determined the CRISPR-Cas12a fluorescence (20 μL) and the test strip reaction system (50 μL). The most obvious band on the strip was the positive band of Fam-TTATT-Biotin, followed by Fam-TCCCT-Biotin and Fam-TTTTTTTATT-Biotin. Therefore, Fam-TTATT-Biotin was chosen for further tests (Figure 4B).

Figure 4C shows the optimization of the reaction time for the RAA-Cas12a assay. It was observed that the reaction tube exhibited noticeable fluorescence after only 15 min of the reaction. The fluorescence intensity gradually increased with the duration of the reaction, as observed using the blue light cutting instrument and the UV glue machine. This indicates that upon detection of the target, the Cas12a protein’s reverse cutting function was activated, leading to continuous cutting of the fluorescence quenching group near the target. As a result, the liquid in the reaction tube became fluorescent.

**Figure 5:**
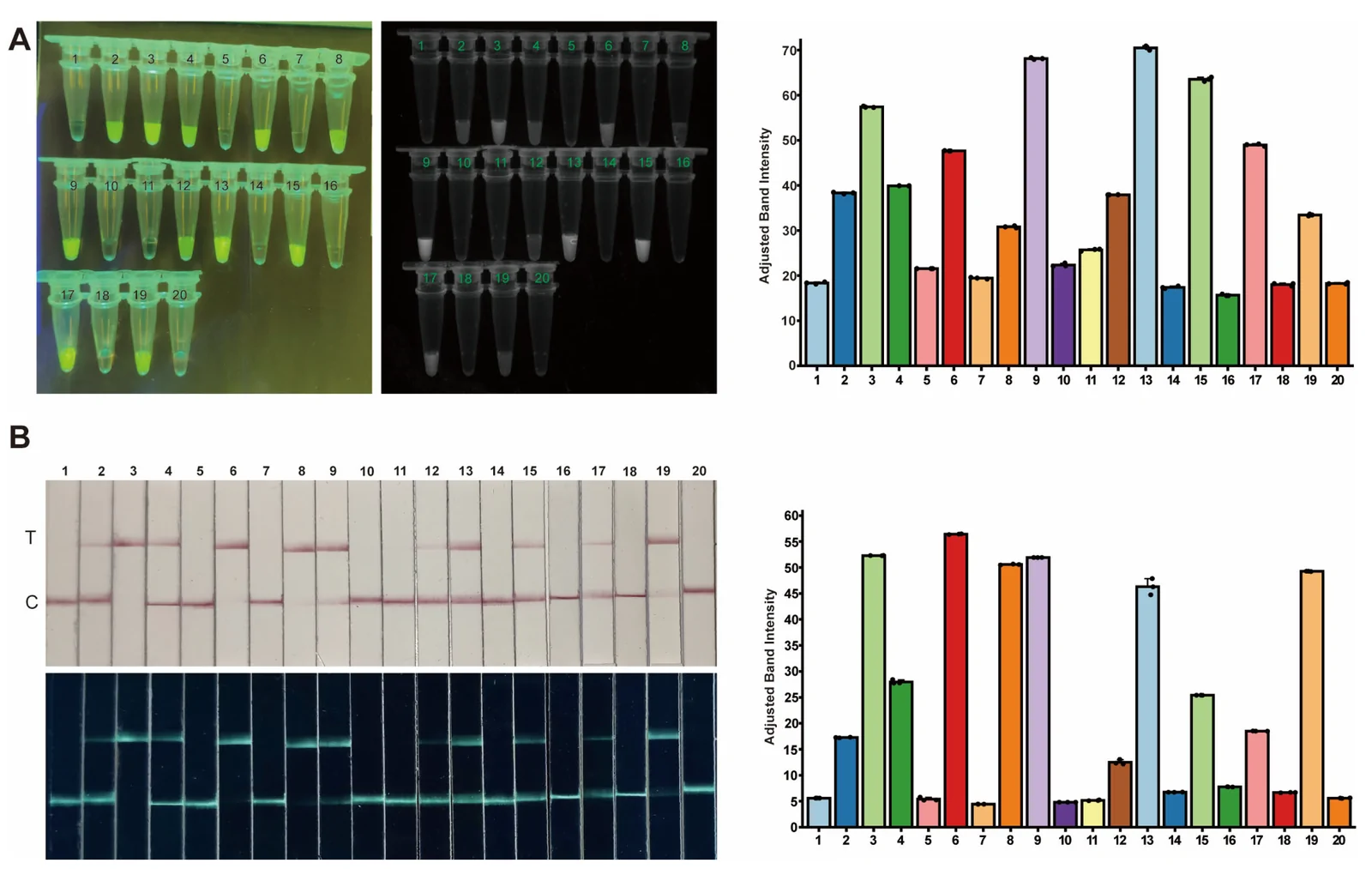
Results of clinical samples of RAA-Cas12a-F (**A**) and RAA-Cas12a-LFD (**B**). The results of the RAA-Cas12a-F assay were analyzed using images obtained under UV light and quantified with ImageJ. N: negative control; C: control line; T: test line.

The color of the T line could only be observed for 15 min. With the increase in reaction time, the intensity of the C line decreased while the intensity of the T line increased. The results of the test strip were strongly positive. The reaction time was 30 min in all subsequent tests. Figure 4D shows that apart from FAdV-4, there was no fluorescence reaction observed in other avian diseases. This indicates that the Cas12a fluorescence method exhibits good specificity. The results of the test strip method were negative for all avian viruses, except for FAdV-4. The sensitivity of RAA-Cas12a-F to FAdV-4 DNA was as low as 10^–6^ ng/μL, and the concentration of RAA-Cas12a-F fluoresced under both blue light and UV irradiation (Figure 4E). The lowest detection limit of the pEASY-T1-ORF19A recombinant plasmid was 10 copies/μL. The lowest detection limit of viral DNA was 10^−5^ ng/μL using the dipstick method. Additionally, the lowest detection limit of plasmid concentration was 100 copies/μL (Figure 4E). By comparing RAA and RAA-LFD, the addition of Cas protein to RAA greatly improved the detection sensitivity. Of the 20 liver clinical samples, 11 samples tested positive for RAA-Cas12A-F and RAA-Cas12A-LFD. The positive rate was 55%, which was consistent with the results of RAA and PCR. The positive coincidence rate was 90.9% (Figure 5).

### 2.4. Detection of Clinical Samples with RAA Related Methods

**Figure 6:**
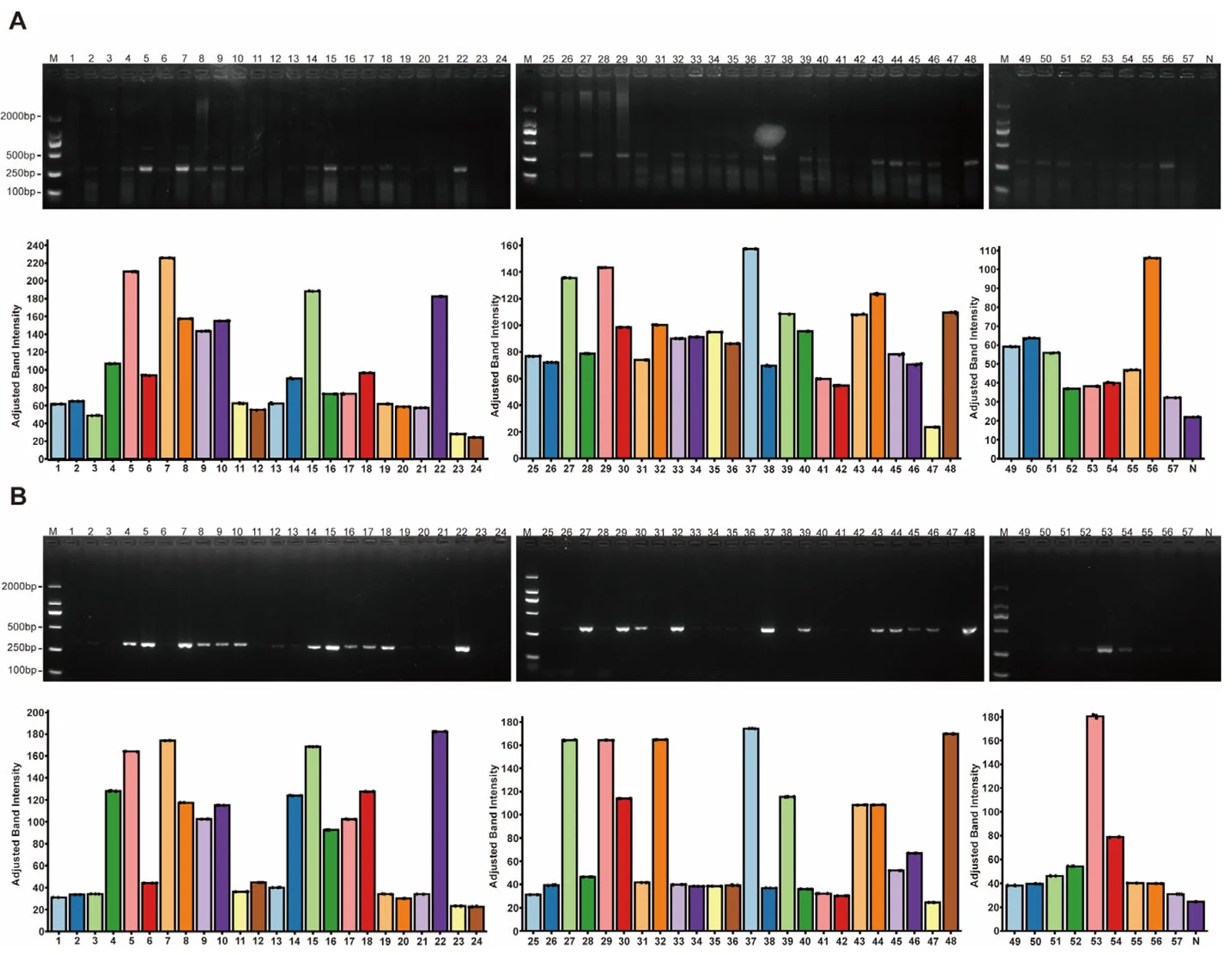
(**A**) Clinical stool samples from Nanping were tested by RAA. (**B**) Clinical stool samples from Nanping were tested by PCR. Lane M: 2000 DNA Marker.

A total of 192 fecal samples from standardized broiler farms in Longyan and Zhangzhou tested negative by the four methods. Additionally, the positive rate of clinical samples collected from large-scale broiler farms in the two cities was 0%. Among the 57 fecal samples collected from free-range chickens in Nanping City, 53 tested positivity, and 53 tested positives using both PCR and RAA. The coincidence rate between the two methods was 100% (Figure 6).

**Figure 7:**
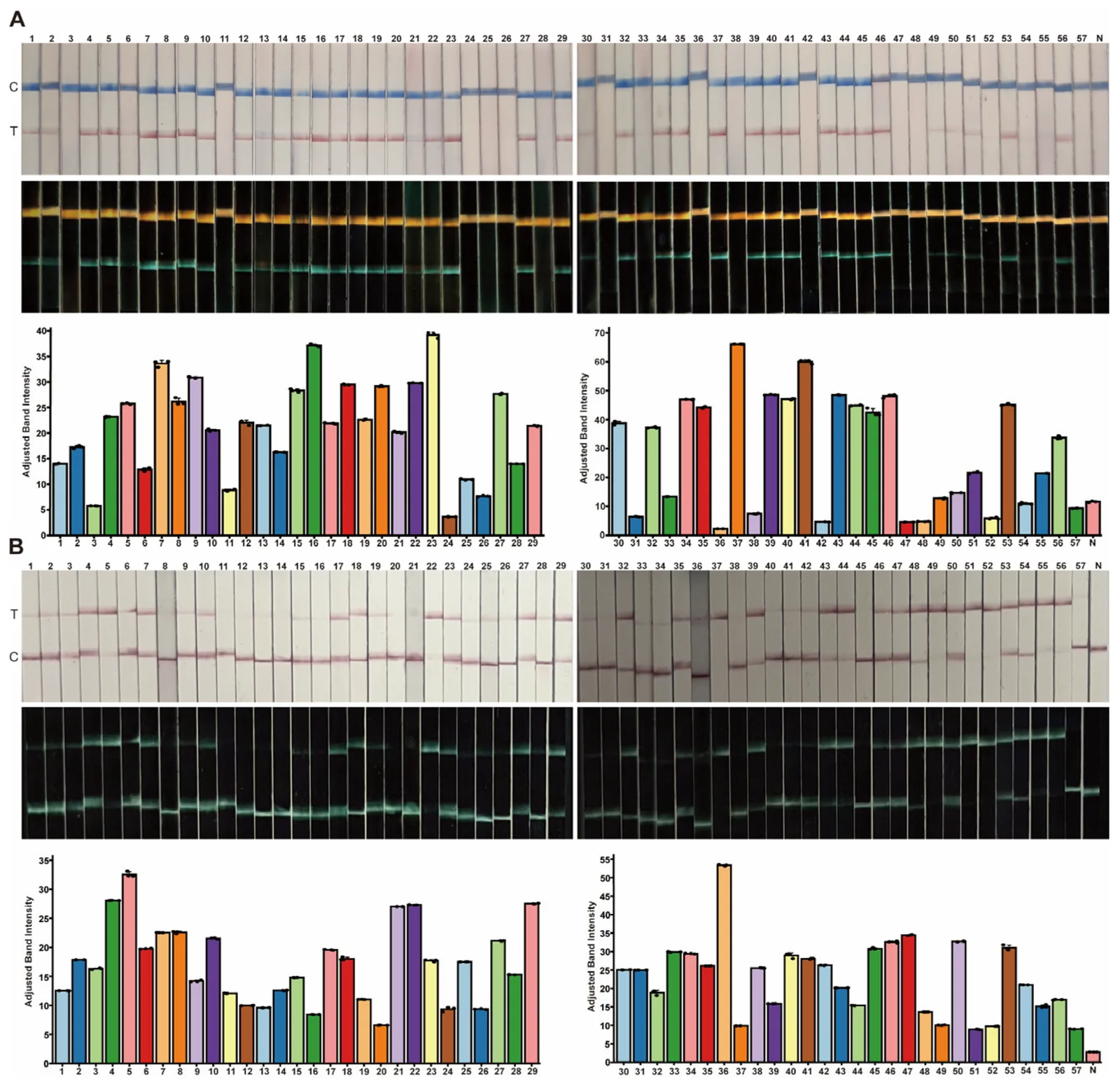
(**A**) Clinical stool samples from Nanping were tested by RAA-LFD. (**B**) Clinical stool samples from Nanping were tested by RAA-Cas12a-LFD. (N: negative control; C: control line; T: test line).

**Figure 8:**
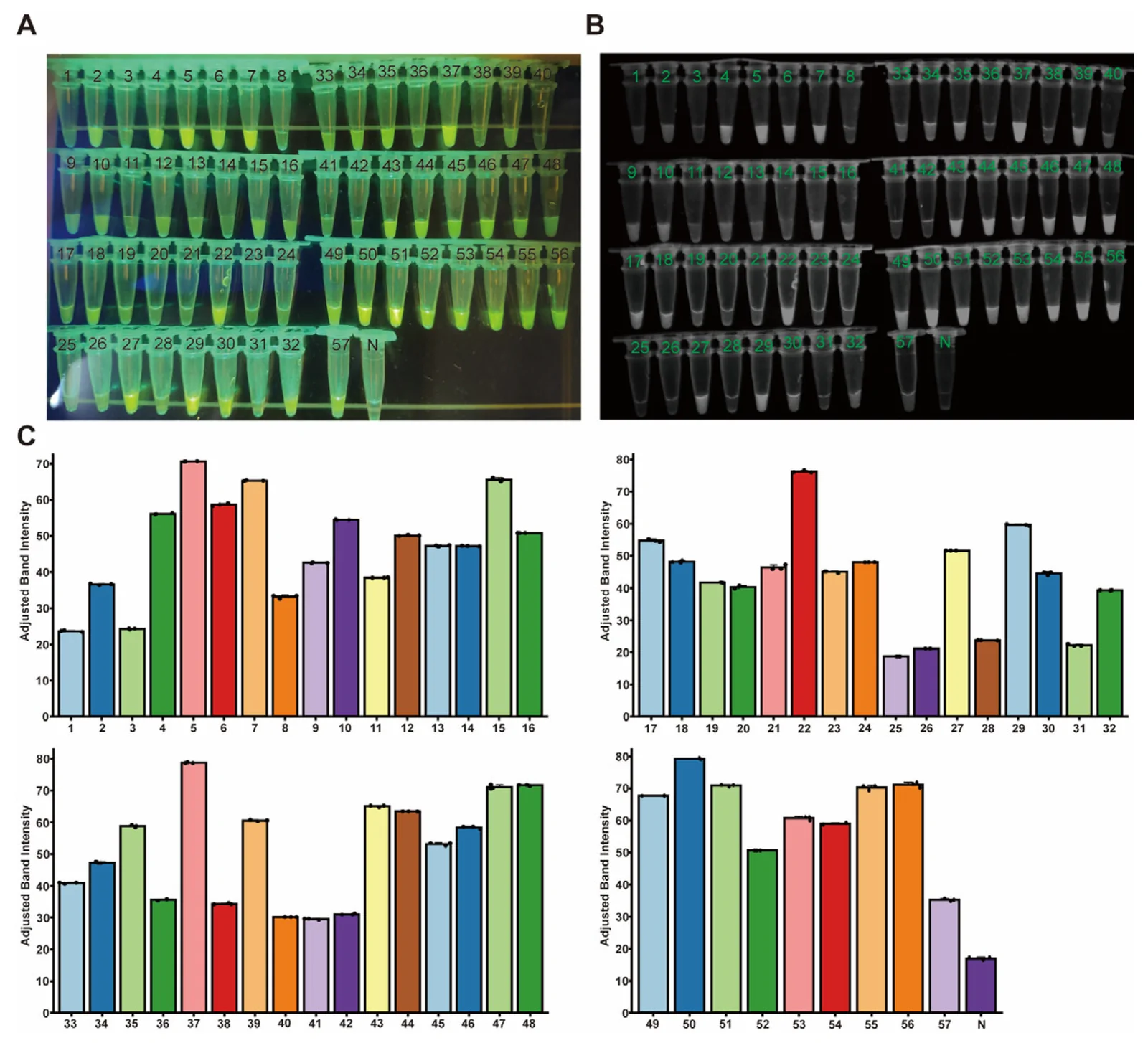
RAA-Cas12a-F was used to detect clinical stool samples in the Nanping area. (**A**,**B**) Results obtained under blue light and UV glue machines, respectively. (**C**) Quantification of images obtained under UV light.

A total of 43 samples were detected positive using RAA-LFD, with a positive coincidence rate of 81.13% (Figure 7A). A total of 57 positive samples were detected by both RAA-Cas12a-F and RAA-Cas12a-LFD methods, resulting in a 100% positive rate for Nanping fecal samples. The positive concordance rate between PCR and RAA-Cas12a-F was 92.98%, and the positive concordance rate between PCR and RAA-Cas12a-LFD was also 92.98%. The positive concordance rate between RAA-Cas12a-F and RAA-Cas12a-LFD was 100%. (Figure 7B and Figure 8). Therefore, RAA-Cas12a with the highest positive rate was determined to be the final result. The total positive rate of FAdV-4 in clinical samples collected from the three cities was 29.69%.

## 3. Discussion

The predominant avian adenovirus serotype found in poultry in China is FAdV-4, which has the capability to infect chickens through both horizontal and vertical transmission [17]. Isolation of multiple FAdV serotypes from the same sick bird is common, suggesting a lack of cross-protection between different FAdV serotypes [18]. However, there is no commercially available vaccine that can prevent FAdV-4 infection. Different species of FAdVs exhibit significant structural and genomic variations [19]. Since there are numerous FAdV serotypes, it is crucial to select specific genes as target sequences for nucleic acid detection [20]. To cope with the characteristics of many serotypes, we invented a series of methods for FAdV based on recombinase-mediated amplification. Compared to the traditional FAdV-4 identification approach, RAA has lower operational requirements and only requires a small thermostatic device set at approximately 39 °C to rapidly obtain numerous nucleic acid fragments. The RAA method utilizes recombinase enzymes derived from bacteria or fungi. The increased availability of these sources has resulted in lower costs compared to the recombinase polymerase amplification (RPA) method [1].

The development of isothermal nucleic acid amplification technologies such as LAMP, RPA, and RAA is progressing rapidly [21]. LAMP is a widely used isothermal nucleic acid amplification technology, and the fluorescent dye method can be used to directly observe the amplification products with the naked eye. The primer design is complicated, requiring 4–6 primers and having strict requirements for the operating environment. Furthermore, the method demands a high-quality operational setting, which limits its application to the detection of target gene presence, with the inability to verify the amplified products through cloning and sequencing [22]. In contrast, the RAA method permits the direct sequencing of the amplicons for validation purposes, offering a robust alternative. The resulting product can then be further confirmed through sequencing to ensure its accuracy. The RAA-LFD assay shortens the time required for nucleic acid amplification, eliminates the need for large-scale temperature-changing equipment, and allows for the presentation of results solely based on test strips, without the need for temperature cycling during the process. It is suitable for rapidly screening diseases in the field, even under poor conditions and with a large number of samples [18]. In this study, the detection of RAA-LFD could be completed within 30 min at a constant temperature of 39 °C, which shortened the detection time by 10 min compared to the RAA method. However, the sensitivity of RAA-LFD in this study was average. This could be attributed to the fixed concentration of commercial test strips of antibody and colloidal gold labeling reagents, which could not be optimized based on the specific amplification products.

The development of the CRISPR-Cas system has opened new avenues for the detection of pathogens through nucleic acid analysis. DNA sensors based on CRISPR-Cas12a technology can detect viral DNA rapidly, reliably, sensitively, inexpensively, and it can selectively detect viral DNA, which is crucial in both industrial and medical applications [23]. Cas12a is a V-type CRISPR protein that cleaves double-stranded DNA and has been used for genome editing [16]. To overcome the disadvantage of low sensitivity, RAA-Cas12a-F and RAA-Cas12a-LFD were established with Cas12a in combination with RAA or RAA-LFD. These combinations significantly improved the sensitivity of nucleic acid detection for FAdV-4. The minimum detectable amount of RAA-Cas12a-F was 10^−6^ ng/μL when using FAdV-4 viral DNA as a template. This is 10,000 times lower than the minimum detectable amount of RAA. Similarly, the minimum detectable amount of RAA-Cas12a-LFD was 10^−5^ ng/μL, which is 1000 times higher than that of RAA. This indicates that the designed FAdV-4 ORF19A-LbaCas12a-crRNA can accurately bind to the target sequence of FAdV-4. The design of Cas12a crRNAs focuses on the presence of a PAM (e.g., the Lba12a TTTV site) upstream of the ssDNA within the target sequence. The ternary complex recognizes the PAM, allowing the downstream sequence to be complementary to the crRNA. The stabilizing interactions with the PAM lead to the formation of a stabilizing R-loop on the complementary substrate, which activates Cas12a for DNA cleavage [24]. At the present study, the upstream region of the amplified target sequence did not contain TTTV sites. Therefore, the TTTA sequence at the 5′ to 3′ end of the downstream sequence was selected as the protospacer adjacent motif (PAM) for the target sequence. The results demonstrated that LbaCas12a maintained efficient cleavage activity when the downstream PAM site was chosen. Additionally, the developed method for detecting FAdV-4 exhibited high sensitivity. However, the RAA-Cas12a for FAdV-4 also has shortcomings. The reaction process requires opening the lid to transfer the RAA reaction product as a template in the system, which increases the risk of aerosol contamination. The RAA-Cas one-tube reaction method is now available. In this method, the RAA reaction reagents and Cas-related reagents are added into one tube at a time, reducing the reaction time and minimizing aerosol contamination [25]. However, the sensitivity of the one-tube method is low, which may be explained by the interaction between the reactive protease in RAA and the Cas-related reagent, affecting the protease activity in the later stage. Whether the one-tube reaction can be carried out by adjusting the concentration of the reagents in the system needs to be investigated. The lack of combination with the nucleic acid-free extraction method prolonged the time it took to detect the virus.

Additionally, when diagnosing clinical samples, the detection of RAA and Cas12a protein binding yields the highest positive rate. The result may be due to the exponential level of amplification of the nucleic acid product through the crRNA in RAA. Following secondary screening, the protein’s cleavage activity is activated, resulting in the cleavage of the fluorescent probe near the target. The cleavage leads to the release of a large number of fluorescent groups, enhancing the visibility of nucleic acid detection. The presence of numerous fluorescent groups enhances the visualization of nucleic acid detection and increases the sensitivity of RAA-Cas12a. In this study, the incidence of FAdV-4 in large-scale chicken farms was much lower than that in free-range farms. It is evident that scalable and standardized farming practices are pivotal in large-scale poultry enterprises for the prevention and control of diseases. These methods encompass various aspects of poultry management, including vaccine prevention and control, as well as the timely diagnosis of diseases in the early stages of breeding to prevent the spread of diseases among poultry.

## 4. Materials and Methods

### 4.1. Virus and Liver Samples

FAdV-4 strain NP, Tembusu virus (TMUV), avian reovirus (ARV) avian leukosis virus (ALV), Newcastle disease virus (NDV) vaccine strain, avian influenza virus H9 subtype, and AIV H9 vaccine strain are stored in our laboratory. Twenty clinical liver samples were obtained from sick chickens that died of suspected FAdV-4 infection. These chickens exhibited signs of hydropericardium, characterized by the accumulation of pale yellow to yellowish fluid in the pericardial sac. Clinical fecal samples were collected from the large-scale broiler farms in Longyan City (*n* = 68), Zhangzhou City (*n* = 67) and free-range broilers in Nanping City (*n* = 57).

All broilers’ experiments were carried out based on the guidelines of the Research Ethics Committee of the College of Animal Science, Fujian Agriculture and Forestry University (NO: PZCASFAFU2016004).

### 4.2. Viral DNA Extraction

The FAdV-4 strain NP (median tissue culture infective dose, TCID50 = 10^−6.23^/0.1 mL) was inoculated into leghorn male hepatocellular (LMH) cells that were cultured in DMEM medium with 5% fetal bovine serum under 5% carbon dioxide conditions. After 24 h, the cells were collected. Viral DNA was extracted using a viral DNA nucleic acid extraction kit (D3892, Omega Bio-Tek Inc., Norcross, GA, USA) following the provided instructions. After grinding and homogenizing the clinical liver samples, the viral DNA was extracted using a DNA nucleic acid extraction kit (DP328, Transgen Biotechnology Co., Ltd., Beijing, China).

### 4.3. The Conserved Genes of FAdV-4 Were Screened and Cloned

The genome sequences of 12 serotypes of FAdV-I were downloaded from NCBI (GenBank accession numbers: A93469.1, NC_001720.1, KT862806.1, KT862805.1, KT862807.1, KU991797.1, MN606302.1, MK757473.1, NC_021221.1, NC_038332.1, KT862809.1, KT862810.1, KT862811.1, MF577036.1, AF083975.2, NC_000899.1, MN542422.1, KT862812.1, Figure S3) and were blasted by Vector NTI alignment software (version 11.5.4, Thermo Fisher Scientific Inc., Waltham, MA, USA). The ORF19A gene (GenBank: KU991797.1) was selected as the target sequence for the RAA assays of FAdV-4. The primers of RAA were designed and are presented in Table 1. The specificity of the primers was determined by PCR using FAdV-4 DNA as the template. The PCR reaction was pre-denatured at 95 °C for 5 min. The PCR protocol consisted of 30 cycles, with denaturation at 95 °C for 30 s, annealing at 58 °C for 30 s, and extension at 72 °C for 30 s. The final extension step was performed at 72 °C for 10 min. The amplified products were purified using an agarose gel DNA recovery kit and then ligated with the pEASY-T1 cloning vector (CT101-01, Transgen Biotechnology Co., Ltd., Beijing, China). The ligation reaction was carried out under the following conditions: 1 μL pEASY-T1+2.1 μL gel recovery product +1.9 μL ddH_2_O. The reaction was performed at 25 °C for 10 min. The ligation product was transformed into Trans1-T1 competent cells (CD501-02, Transgen Biotechnology Co., Ltd., Beijing, China). The recombinant plasmid was extracted by a plasmid kit (DP118, Transgenic Biotechnology Co., Ltd., Beijing, China) for identification by PCR. The PCR product was sent to Platinum Biotechnology Company (Fuzhou, China) for sequencing. The concentration of recombinant plasmid was determined by NanoDrop 2000. The concentration of plasmid was calculated by the formula for copy number: plasmid copy number = [(6.02 × 10^23^) × (concentration ng/μL × 10^−9^)]/(DNA base number bp × 660).

### 4.4. The RAA Reaction System Was Established

Distinguished from PCR primer design, RAA primers are typically 30–35 bp in length, longer than conventional PCR primers [18]. In this study, nine pairs of specific RAA primers were designed and synthesized based on these principles using Oligo 7 software (Table 1). Then, the most suitable primers were selected through RAA agarose gel electrophoresis. The primers were synthesized by TSINGKE Biological Technology, Beijing, China. The isothermal amplification was performed with a commercial RAA kit (B00000, Jiangsu Qitian Gene Biology Co., Ltd., Wuxi, China). RAA assays were performed in a 50-μL system with the following components: 18.5 μL of sterile water, 25 μL of buffer, 2 μL each of forward and reverse primers (10 μmol/L), and 2.5 μL of magnesium acetate (280 mmol/L). All ingredients, except magnesium acetate were added to the reaction tube. Prior to this, the reaction tube contains 800 ng/μL SSB, 120 ng/μL uvsX, and 30 ng/μL DNA polymerase. The reaction systems mentioned above were mixed and then placed in either a water bath, PCR instrument, or constant-temperature incubator. After the reaction, 50 μL of DNA extract (T0250, Beijing Solaibao Technology Co., Ltd. Beijing, China) was added into each tube, shaken thoroughly, and centrifuged at 13,400 rpm for 1 min. The upper solution was aspirated and combined with the reaction buffer. It was then subjected to 2% agarose gel electrophoresis at 220 V for 20 min. Finally, the gel was placed in a gel imager to observe the results. 

Using FAdV-4 viral DNA as a template, RAA amplification was performed at temperatures of 35 °C, 37 °C, 39 °C, 41 °C, and 43 °C to determine the optimal reaction temperature for subsequent reactions. Based on the optimal reaction temperature, RAA amplification was performed at different time intervals (10, 20, 30, 40, and 50 min) to determine the optimal reaction time.

### 4.5. Evaluation of RAA for FAdV-4

Firstly, the sensitivity determination was carried out. The extracted FAdV-4 DNA was prepared at a concentration of 100 ng/μL and then diluted to various concentrations. Samples with concentrations of 10^1^, 10^0^, 10^−1^, 10^−2^, 10^−3^, and 10^−4^ ng/μL were obtained for RAA and PCR amplification, respectively. PCR 50 μL reaction system: 25 μL of 2×SanTaq PCR Master Mix (B53206, Shanghai Sangon Biological Co., Ltd., Shanghai, China), 2 μL each of upstream and downstream primers (10 μm), 19 μL of ddH2O, and 2 μL of template. The PCR reaction was pre-denatured at 95 °C for 5 min. There were 35 cycles of denaturation at 95 °C for 30 s, annealing at 58 °C for 30 s, and extension at 72 °C for 30 s. The final extension was carried out 72 °C for 10 min. Then, the recombinant plasmids were diluted by 10-fold gradient, obtaining different concentrations (10^5^–10^0^ copies/μL) as templates for RAA detection.

Secondly, we evaluated the specificity. The cDNA of ALV, ARV, AIV H9, TMUV, and NDV and the DNA of FAdV-4 were used as templates for RAA detection to determine the specificity of RAA assay. Specificity testing for fowl adenovirus 10 (FAdV-10, MN542422.1) is detailed in Figure S4.

Thirdly, repeatability tests were conducted. Three reaction tubes in the same kit were used for three intra-batch repeatability tests on positive samples. Additionally, three reaction tubes from two kits produced at different times were used for three inter-batch repeatability tests of positive samples.

Finally, viral DNA was extracted from the livers of 20 chickens suspected to be infected with FAdV-4. The extracted DNA was then subjected to RAA and PCR amplification, respectively. The rates of positive coincidence for the two methods were calculated.

### 4.6. Establishment and Development of RAA-LFD

In order to facilitate the presentation of the results of RAA, we designed a nucleic acid probe based on the production of RAA. The upstream primer of RAA-LFD was the same as RAA. The downstream primer was labeled with biotin at the 5′ end (Table 1). The probe was located in the conserved and specific part of the nucleic acid amplification product, labeled with the carboxyfluorescein (FAM) group at the 5′ end (Figure S2). A tetrahydrofuran (THF) debasing agent was added internally, and a C3-spacer blocker was added at the 3′ end. The primers and probe mixtures were diluted to 10 μM, 5 μM, 2.5 μM, 1.25 μM, 0.625 μM, and 0.3125 μM, respectively. RAA-LFD was then performed to determine the optimal concentration. A 50 μL amplification system was prepared according to the instructions provided with the RAA nucleic acid amplification kit (dip-strip method). The disposable nucleic acid test strip (JY0201) was obtained from Beijing Baoying Tonghui Biotechnology Co., Ltd. (Beijing, China).

RAA-LFD reaction system: buffer, 25 μL; sterile water, (17.7-x) μL; forward and reverse primers, 2.1 μL each; probe, 0.6 μL; magnesium acetate (280 mmol/L), 2.5 μL; template, x μL. After the reaction, the reaction tube was removed, and 10 μL of the amplification product was transferred and mixed with 90 μL of 1×PBS in a 1.5 mL EP tube. The test strip’s binding pad was placed into the EP tube for incubation, and the results were read within 5 min. A clear detection line (T) and a quality control line (C) were formed on the LFD strip. They indicated that the samples contain nucleic acid fragments to be detected. In addition, only quality control lines were observed in the samples of negative controls. The pEASY-T1-ORF19A recombinant plasmid was used as a template to screen the optimal reaction conditions in terms of temperature and time. Each 1 °C represented a gradient within the range of 37 °C to 41 °C, respectively. In addition, RAA-LFD amplification was performed at the optimal reaction temperature for different time gradients (10, 20, 30, 40, and 50 min).

### 4.7. Evaluation of RAA-LFD Test

The sensitivity of the RAA-LFD reaction system was determined by diluting FAdV-4 viral DNA to concentrations of 100, 50, 25, 12.5, and 6.25 ng/μL, followed by RAA amplification. The gradient dilution of the recombinant plasmid was the same as that of the RAA method. The specificity, repeatability, and clinical detection of liver samples were the same as those with the RAA method. The results of amplification were observed by test strips.

### 4.8. CrRNA Preparation

The lower sensitivity of the RAA-LFD will impact its application. The LbaCas12a-crRNA was designed based on the RAA-amplified sequence. The PAM identified by Cas12a was 5′-TTTV-3′, where V represented for A/G/C. The PAM was recognized by the fixed repeat sequence of LbaCas12a (EZassay Biotech Co. Ltd., Shenzhen, China). After performing PAM, the fixed repeat sequence and the spacer sequence of 20–23 nt were combined to create LbaCas12a-crRNA. RAA primers should not overlap with the spacer sequence, and the selected spacer sequence was analyzed by BLAST (https://blast.ncbi.nlm.nih.gov/Blast.cgi, accessed on 18 February 2025) to determine its conservation and specificity. The ssDNA of crRNA was synthesized by TSINGKE Biological Technology (Beijing, China). The design of LbaCas12a-crRNA is shown in Table 1. The synthesis steps for FAdV-4-LbaCas12a-crRNA are as follows: The ssDNA of crRNA was used as a template, and the corresponding primers were amplified by PCR to obtain a large quantity of dsDNAs of crRNA. The target band was recovered by the Gel Extraction Kit (CWBIO Technology Co., Ltd., Taizhou, China) and purified with the HiScribe T7 Quick High Yield RNA Synthesis Kit (New England Biolabs, Ipswich, MA, USA). The transcripts were purified by the RNA Purification Kit (DP412, Tiangen Biochemical Technology Co., Ltd., Beijing, China) and stored at −80 °C after measuring the RNA concentration with Nanodrop 2000. PCR reaction system (25 μL): 2× SanTaq PCR Master Mix (with Blue Dye), 12.5 μL; crRNA-dsDNA, 1 μL; upstream and downstream primers, 1 μL each (10 μM); ddH2O, 9.5 μL. The PCR reaction procedure was the same as the RAA reaction, except that the annealing temperature was adjusted to 56 °C. Transcription system: T7 mix, 2 μL; NTP mix, 10 μL; template, 1 μg. ddH_2_O was added to make a total volume of 20 μL.

### 4.9. The RAA-Cas12a-F and RAA-Cas12a-LFD Reaction Systems Were Established

Based on the study of RAA and Cas12a probe base preferences, Fam-TCCCT-BHQ was used as the probe for RAA-Cas12A-F. RAA-Cas12a-LFD was performed in a 50 μL system by comparing the effects of three probes: Fam-TCCCT-Biotin, Fam-TTATT-Biotin, and Fam-TTTT-Biotin. At the end of the reaction, the test strip was inserted into the reaction centrifuge tube, and the results were evaluated within 5 min. CrRNA concentrations were set at 100 nM, 200 nM, 300 nM, 400 nM, and 500 nM, and they were reacted with Cas12a protein at 50 nM.

The reaction system specifically included 10 × reaction buffer, rRNA (10 μM), and a probe (10 μM); template (0.8 μg) of LbaCas12a protein, 1 μM, was mixed with 15.2 μL of ddH2O. The optimal reaction system was the CRISPR-Cas12a reaction system. In addition, the reaction time was set as 15, 20, 25, and 30 min, and the results were read using blue light, ultraviolet light, and a test strip, respectively. In addition, the disposable nucleic acid test strip (JY0301) was purchased from Beijing Baoying Tonghui Biotechnology Co., Ltd.

The DNA of FAdV-4 and the pEASY-T1-ORF19A recombinant plasmids were used as templates to determine the sensitivities of RAA-Cas12a-F and RAA-Cas12a-LFD. FAdV-4 DNA was diluted by 10-fold gradient, obtaining 10^−1^–10^−7^ng/μL for testing. After RAA amplification, the pEASY-T1-ORF19A recombinant plasmids were diluted from 105 to 10^−2^ copies/μL in a 10-fold gradient, and the results were observed by ultraviolet (UV) light and a dipstick. The specificity of amplification was tested in liver clinical samples using the same method as described above for the RAA method. The results were read using UV and dipstick.

### 4.10. Extraction and Monitoring of Fecal Genomic DNA

Genomic DNA was extracted from clinical stool samples. Fecal genomic DNA extraction (DP328, Tiangen Biochemical Technology Co., Ltd.): After incubating at 70 °C for 15 min, the supernatant was centrifuged and then added to the adsorption column collection tube. Buffer and rinse solution were added to the waste solution after centrifugation, and 50 μL of eluate was collected to obtain DNA. It was stored at −20 °C until used. Clinical stool samples were detected using the constructed RAA, RAA-LFD, and RAA-Cas12a-LFD methods.

### 4.11. Figure Preparation

To quantify and visualize the FAdV-4 DNA amount in the test sample, the band density was measured by ImageJ v1.53c. To homogenize the image, all bands were converted to grayscale for sampling purposes, and the sample band lane with rectangular selections. Then plot lanes to create a profile plot for each lane, which represents the relative density of the target band. Then, the band density was prepared using Chiplot (https://www.chiplot.online). The figures were assembled in Adobe Illustrator. Test flow chart components were assembled from www.figdraw.com.

## 5. Conclusions

**Figure 9:**
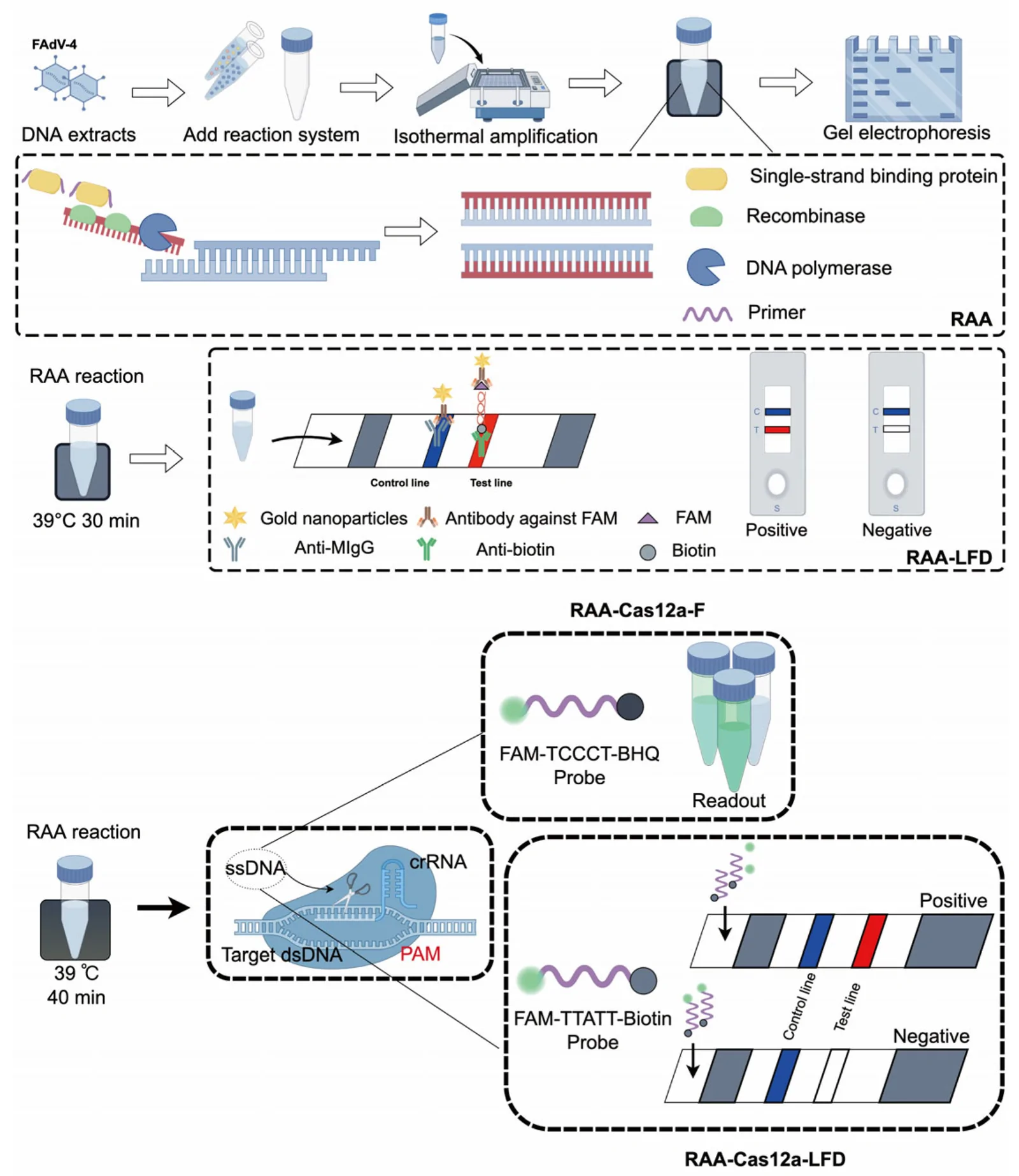
Schematic diagram of the RAA, RAA-LFD, RAA-Cas12a-F and RAA-Cas12a-LFD detection systems. Created with Figdraw.com.

In this study, we developed four different RAA methods for rapid and accurate FAdV-4 diagnosis (Figure 9). Among these methods, the RAA-LFD of FAdV-4 is slightly less sensitive but offers the advantages of visualization, convenience, and speed. Both RAA-Cas12a-F and RAA-Cas12a-LFD demonstrate higher sensitivity and specificity. In summary, the methods may make a significant contribution to FAdV-4 control.

## Figures and Tables

**Table 1:** Primers and probes used in this work.

Genes Name	Prime Name	Sequences (5’-3’)	Genomic Position (KU991797.1)	Product Size (bp)
ORF19A (GenBank: KU991797.1)	RAA-F1	CTACAACTACTCCCACCACTACAACCACTACTCC	42,092–42,125	349
	RAA-R1	GTACCTGGGGATACGGTGAGTTGAAGAG	42,413–42,440	
	RAA-F2	CTACAACTACTCCCACCACTACAACCACTACTCC	420,92–42,125	446
	RAA-R2	AATGGTGACAGGAATGGTGACCGTGGAGT	42,509–42,537	
	RAA-F3	CTACAACTACTCCCACCACTACAACCACTACTCC	420,92–42,125	494
	RAA-R3	AATGAATACGGCAATGATAACACTGAGACAGAC	42,553–42,585	
	RAA-F4	CACTACTCCCACTACGACCACGTCCTCCA	42,186–42,224	303
	RAA-R4	GTACCTGGGGATACGGTGAGTTGAAGAG	42,461–42,488	
	RAA-F5	CACTACTCCCACTACGACCACGTCCTCCA	42,186–42,224	400
	RAA-R5	AATGGTGACAGGAATGGTGACCGTGGAGT	42,557–42,585	
	RAA-F6	CACTACTCCCACTACGACCACGTCCTCCA	42,186–42,224	448
	RAA-R6	AATGAATACGGCAATGATAACACTGAGACAGAC	42,601–42,633	
	RAA-F7	AACAATGAAACCTCAGAACCAACTACTGCTGCTAC	42,337–42,371	143
	RAA-R7	GTACCTGGGGATACGGTGAGTTGAAGAG	42,451–42,479	
	RAA-F8	AACAATGAAACCTCAGAACCAACTACTGCTGCTAC	42,337–42,371	240
	RAA-R8	AATGGTGACAGGAATGGTGACCGTGGAGT	42,548–42,576	
	RAA-F9	AACAATGAAACCTCAGAACCAACTACTGCTGCTAC	42,337–42,371	297
	RAA-R9	AATGAATACGGCAATGATAACACTGAGACAGAC	42,601–42,633	
	RAA-Probe	FAM-TCCAGCACTGCCATCATAGAGGAAGAAGAA(THF)ACAGC ACGGAACCGA-C3-spacer	42,380–42,408	
	RAA-LFD-F	AACAATGAAACCTCAGAACCAACTACTGCTGCTAC	42,337–42,371	
	RAA-LFD-R	Biotin-AATGAATACGGCAATGATAACACTGAGACAGAC		
LbaCas1	LbaCas12acrRNA-F	*TAATACGACTCACTATAGGG*TAATT		
2acr RNA	LbaCas12acrRNA-R	TTCCTCTGGGCGGAACCATCATC		

Note: The T7 promoter sequence is in italics; underlined bases indicate repetitive sequence regions.

## Data Availability

The data presented in this study are available on request from the corresponding author.

## Supplementary Materials

The following supporting information can be downloaded at: https://www.mdpi.com/article/10.3390/ijms27219756/s1.
