# Supplementary material for: Four Recombinase-Aided Amplification Assays for the Rapid Detection of Fowl Adenovirus Serotype 4
Source: Int J Mol Sci. 2026 Oct 30;27(21):9756. doi: 10.3390/ijms27219756 (PMC13763254; doi:10.3390/ijms27219756)
Supplement: Supplementary file 1 [file ijms-27-09756-s001.zip › Supplementary data.pdf]

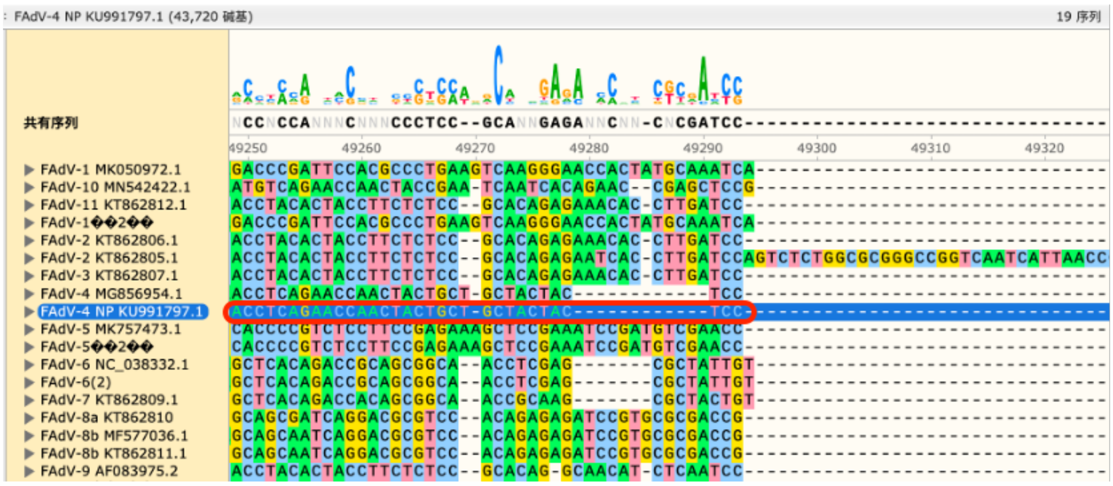

Figure S1 The ORF19A target sequence

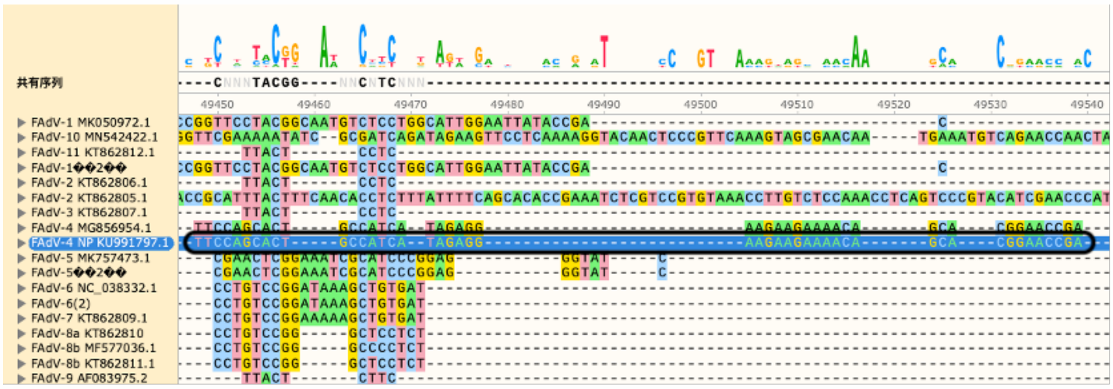

Figure S2 The probe utilized in the RAA-LFD method

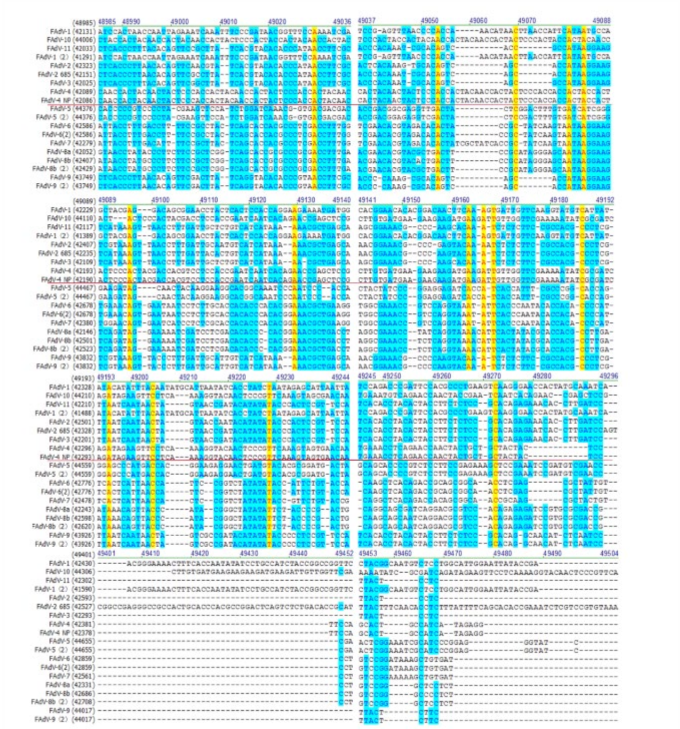

Figure S3 The genomes of 12 FAdV-I serotypes were relatively conserved and specific

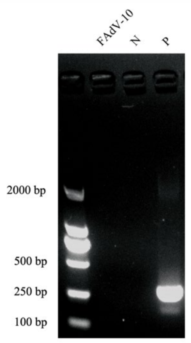


Figure S4 The validation of FAdV-10 specificity. lane N: Negative control. lane P: Positive control.

Below are the primer sequences used for FAdV-10 detection in our optimized RAA assay:

Forward primer (F): 5′ -AAAAGGTACAACTCCCGTTCAAAG-3′

Reverse primer (R): 5′ -TCTAAGGGCAATGAATACGGCAAC-3′

Amplicon size: 308 bp

These primers were specifically designed to target the Fowl adenovirus 10 (FAdV-10, MN542422.1) genome, ensuring no cross-reactivity with other serotypes (e.g., FAdV-4).
